# Validation of patient determined disease steps (PDDS) scale scores in persons with multiple sclerosis

**DOI:** 10.1186/1471-2377-13-37

**Published:** 2013-04-25

**Authors:** Yvonne C Learmonth, Robert W Motl, Brian M Sandroff, John H Pula, Diego Cadavid

**Affiliations:** 1 Department of Kinesiology and Community Health, University of Illinois at Urbana-Champaign, Urbana, IL, USA; 2 College of Medicine, University of Illinois at Peoria, Peoria, IL, USA; 3 Illinois Neurological Institute, Peoria, IL, USA; 4 Biogen Idec, Weston, MA, USA

## Abstract

**Background:**

The Patient Determined Disease Steps (PDDS) is a promising patient-reported outcome (PRO) of disability in multiple sclerosis (MS). To date, there is limited evidence regarding the validity of PDDS scores, despite its sound conceptual development and broad inclusion in MS research. This study examined the validity of the PDDS based on (1) the association with Expanded Disability Status Scale (EDSS) scores and (2) the pattern of associations between PDDS and EDSS scores with Functional System (FS) scores as well as ambulatory and other outcomes.

**Methods:**

96 persons with MS provided demographic/clinical information, completed the PDDS and other PROs including the Multiple Sclerosis Walking Scale-12 (MSWS-12), and underwent a neurological examination for generating FS and EDSS scores. Participants completed assessments of cognition, ambulation including the 6-minute walk (6 MW), and wore an accelerometer during waking hours over seven days.

**Results:**

There was a strong correlation between EDSS and PDDS scores (*ρ* = .783). PDDS and EDSS scores were strongly correlated with Pyramidal (*ρ* = .578 &*ρ* = .647, respectively) and Cerebellar (*ρ* = .501 &*ρ* = .528, respectively) FS scores as well as 6 MW distance (*ρ* = .704 &*ρ* = .805, respectively), MSWS-12 scores (*ρ* = .801 &*ρ* = .729, respectively), and accelerometer steps/day (*ρ* = -.740 &*ρ* = -.717, respectively).

**Conclusion:**

This study provides novel evidence supporting the PDDS as valid PRO of disability in MS.

## Background

The monitoring of disease progression among persons with multiple sclerosis (MS) in clinical research and practice has typically been undertaken with the Kurtzke [1] Expanded Disability Status Scale (EDSS) [2]. Based on its shortcomings, researchers developed the Disease Steps (DS) as a simple and reproducible assessment of disability that can be undertaken by neurologists who are not specialists in MS [3,4]. The DS was developed based on motor and ambulatory dysfunction representing the main recognizable features of MS disability [3,4]. Researchers initially reported a high concordance in ranking of subjects between DS and EDSS scores (*ρ* = .958), and substantial agreement in DS scores between raters (*κ* = .80) [3]. The same researchers later confirmed the high concordance in rankings (*ρ* = .944), and reported correlations between changes in DS and EDSS scores over 1 (*ρ = .545*), 2 (*ρ = .635*), and 3 (*ρ = .626*) year periods [4]. Other researchers further reported that the EDSS and DS correlated similarly and reasonably with patient-reports of neurological impairment, disability, and quality of life [5].

Researchers associated with the Patient Registry of the North American Research Committee on MS (NARCOMS) later developed the Patient Determined Disease Steps (PDDS) scale as a patient-reported outcome (PRO) of disability in MS [6]. The PDDS was adapted from the physician administered DS [3,4] to be a surrogate of the EDSS [7]. The PDDS has nine ordinal levels ranging between 0 (normal) and 8 (Bedridden) [8] and PDDS scores can be converted into EDSS scores [9] as well as classifications of mild, moderate, or severe disability [10]. We do recognise that the PDDS and EDSS scales are not isomorphic, and there is not a direct correspondence between scores. Nevertheless, the PDDS scale has been included in research on spasticity [6], treatment patterns [8], pain [11], fatigue [12], employment [13], comorbidities [14,15], quality of life [16], and physical activity [17], for example, in persons with MS.

To date, there has been limited research on the actual validity of PDDS scores, despite its sound conceptual development and inclusion in diverse studies of MS. We are aware of only one study that reported a correlation between PDDS and EDSS scores (*ρ* = .64) in 44 persons with MS who had a median EDSS score of 3.5 [7]; that correlation is not consistent with the often reported correlation coefficients of .93 [11] and .958 [9] for the validity of the PDDS. The first correlation cannot be located for verification in the frequently cited paper [5], whilst the second seemingly represents the correlation between DS and EDSS scores [3]. Other researchers have reported associations between PDDS scores and ambulation outcomes such as Multiple Sclerosis Walking Scale-12 (MSWS-12) scores (*ρ = .847)*, six-minute walk (6 MW) performance (*ρ = -.427)*, and free-living accelerometry (*ρ = .519)* in 26 persons with MS [18]; this too provides limited evidence for the validity of the PDDS, as this scale is heavily dependent upon ambulation much like the DS and EDSS.

Overall, the PDDS is a promising PRO of disability that is simple and economical compared with the EDSS and DS and this scale has the potential to be used for the assessment of disability in clinical practice and in clinical trials. Yet, the current evidence for the validity of PDDS scores is minimal and often misreported. To that end, this study (1) examined the bivariate association between PDDS and EDSS scores as an approach for providing evidence of criterion validity for the PDDS and (2) compared the bivariate associations between PDDS and EDSS scores with Functional System (FS) scores, ambulatory outcomes, cognitive processing speed, and clinical and demographic factors in persons with MS as an approach for providing evidence of convergent and divergent aspects of construct validity for the PDDS.

## Methods

### Sample

The sample was recruited through neurology practices located in the USA, and testing occurred in a single MS center. The three inclusion criteria were (a) neurologist confirmed diagnosis of MS [19] (b) capacity for independent ambulation or ambulation with an assistive device and (c) willingness to voluntarily complete testing. Those who had a relapse in the past 30 days were excluded from participation. Participants were recruited through an email flyer that was distributed among participants in a database from previous studies conducted in the laboratory over the past five years and through local media, promotional flyers and medical records. Overall, 190 people were contacted, 124 were screened and recruited, but 28 cancelled and were unable to be re-scheduled. The final sample included 96 patients who satisfied inclusion criteria and participated.

### Measures

#### Timed 25-foot walk (T25FW)

The T25FW was administered as a measure of walking speed. This assessment consisted of the participant walking 25 feet, with or without an assistive device, as quickly and safely as possible in a hallway clear of obstacles. The main outcome measure was the mean time (s) to complete two trials of the T25FW [20]; shorter times reflect faster walking speed.

#### Timed-Up-and-Go (TUG)

The TUG was administered as a measure of walking mobility [21] as it involves standing up, walking, turning, and sitting down. The TUG consisted of the participant sitting on a chair, standing up with arms crossed over the chest, walking around an object placed 10 feet in front of the chair, and returning to a seated position on the chair as quickly and safely as possible. The participants completed two trials and the main outcome was mean time (s) to complete the TUG [21] (i.e., time from arising from the chair to the moment sitting back on the chair); shorter times reflect better walking mobility.

#### Six-minute walk

The 6 MW was included as a measure of walking endurance. The 6 MW was performed using a rectangular and carpeted course with four hallways that each exceeded 50 m in length and that were clear of obstructions and foot traffic. The participants walked around the entire course during the 6 MW in a clockwise pattern. We provided standardised instructions and emphasised walking as far and as fast as possible for 6 minutes [22]. Distance was recorded in meters (m) using a measuring wheel (Stanley MW50, New Briton, CT); longer distances reflect better walking endurance.

#### Multiple sclerosis walking scale-12

The MSWS-12 is a 12-item PRO of the impact of MS on walking [23]. Example items are “In the past two weeks, how much has MS limited your ability to walk?” and “In the past two weeks, how much has MS slowed down your walking?” The 12-items on the MSWS-12 are rated on a scale ranging between 1 (*Not at all*) and 5 (*Extremely*). The total MSWS-12 score is computed by summing the individual item scores, subtracting the minimum possible score (12), dividing by the maximal score (48), and then multiplying the result by 100 [23]. The MSWS-12 score ranges between 0 and 100; lower scores indicate less perceived walking impairment.

#### Abbreviated Late-Life Function and Disability Inventory (LL-FDI)

The abbreviated LL-FDI is a multidimensional, PRO of functional limitations and disability with psychometric evidence of validity in persons with MS [24]. The functional limitations component of the abbreviated LL-FDI was included in this study and contains 15 items partitioned into three, five-item subscales, namely advanced lower extremity function (ALEF), basic lower extremity function (BLEF), and upper extremity function (UEF). An example item for the ALEF subscale was “How much difficulty do you have with going up and down a flight of stairs outside, without using a handrail?” An example item for the BLEF subscale was “How much difficulty do you have using a step stool to reach into a high cabinet?” An example item for the UEF subscale was “How much difficulty do you have unscrewing the lid off a previously unopened jar without using any devices?”. The 15-items were rated on a 5-point ordinal scale of 1 (none) and 5 (cannot do) and were reverse-scored (i.e., 1 was re-coded into 5, whereas 5 was recorded into 1) and then averaged to form composite measures of ALEF, BLEF, and UEF. Scores for each five-item subscale range between 5 and 25, and higher scores reflect fewer functional limitations.

#### Free-living accelerometry

ActiGraph accelerometers (model GT3X; ActiGraph) measure steps/day as an indication of free-living ambulation in MS [25]. The ActiGraph accelerometers were worn on a belt over the hip and measured steps using a solid state digital accelerometer that generates an electrical signal proportional to the force acting upon it during movement. The steps were recorded over one-minute intervals, stored in the accelerometer’s memory and later downloaded using a personal computer. Steps per one-minute interval were summed over the course of the day into steps/day. Raw accelerometer data were checked against participant recorded wear times from a log sheet and only valid days (≥ 10 hours of wear time without periods of continuous zeros exceeding 60 minutes) were included in the analysis. The outcome of steps/day was averaged over 3 or more available days of data, and higher scores reflect greater community ambulation.

#### Cognitive processing speed

The 3-second Paced Auditory Serial Additional Test (PASAT) and the Symbol Digit Modalities Test (SDMT) were included as measures of cognitive processing speed. These tests are relatively quick assessments and valid in MS [26,27]. The PASAT emphasises auditory processing speed and working memory, whereas the SDMT involves visual/spatial processing speed and working memory; detailed procedures for the PASAT and SDMT are provided elsewhere [28]. The main outcome measure of the PASAT was the total number of correct responses given out of a possible 60 [26]. The main outcome measure of the SDMT was the total number of correctly provided numbers (maximum of 110) in the 90 second period [27]. Higher scores on both assessments reflect faster cognitive processing speed.

### Procedure

The procedure was approved for human subjects research by the University of Illinois College of Medicine at Peoria Institutional Review Board and all participants provided written informed consent. The data were collected from each participant during one session in a single clinical setting. There was no standardization of the exact ordering of tests as more than one person underwent testing per session. Rather, we varied the administration of tests such that there was ample seated rest between the administration of walking outcomes (i.e., each walking outcome was followed by a seated rest period and administration of a non-ambulatory outcome as an approach for avoiding motor fatigue). The participants provided demographic information, completed the PDDS, MSWS-12, and LL-FDI, and underwent a neurological examination for generating FS and Expanded Disability Status Scale (EDSS) scores [1]. This was accompanied by completion of the SDMT and PASAT, T25FW, TUG, and 6 MW tests. The participants were then provided with an accelerometer, belt, log, and instructions for wearing the motion sensor during the waking hours of the next seven days, along with a pre-stamped and pre-addressed envelope for its return. All participants received $20 remuneration upon return of the motion sensor.

### Validation framework

To establish criterion validity, the correlation between PDDS and EDSS scores were examined as the EDSS is the most common and accepted measure of disability status in MS. To establish the convergent and divergent aspects of construct validity, we examined the correlations between PDDS scores with FS scores and other clinical outcomes. The correlations with measures related to mobility (i.e., pyramidal functions, cerebellar functions, sensory functions, 6 MW, T25FW, TUG, steps/day, BLEF and ALEF) provided information on the convergent validity of the PDDS, whilst comparisons with outcomes related to other, non-mobility constructs (i.e. optic functions, brainstem functions, bowel/bladder functions, mental status function, demographic variables, UEF, SDMT and PASAT) provide information on the divergent validity of the PDDS.

### Data analysis

The data were analysed using IBM SPSS statistics version 19.0. Descriptive statistics were computed as median (range, IQR), unless otherwise noted. The associations between variables were examined using Spearman rho rank-order correlation coefficients (*ρ*) given that the EDSS and PDDS are both ordered-categorical variables. This approach further avoids the effects of outliers and non-normality on the correlation coefficients [29]. Values for correlation coefficients of .1, .3, and .5 were interpreted as small, moderate, and large, respectively [30]. We examined the significance of differences in the magnitude of dependent correlations between PDDS and EDSS scores with other variables [31]; the significance of differences was based on an alpha value of .05.

## Results

### Demographic and clinical characteristics of sample

The demographic characteristics of the 96 patients with MS are provided in Table 1. The sample had a median (IQR) age of 53.5 (14) years and was mostly female (*n* = 77 or 80%). The sample consisted of participants who were well educated (43% had a college education) and 59 percent earned $40,000 or more annually. The sample consisted of mostly individuals with relapsing remitting MS (82%) who had been diagnosed for a median (IQR) duration of 9 (12) years.

**Table 1:** Demographic and clinical characteristics of the 96 multiple sclerosis patients

**Variable**	**Descriptive statistic**
Age (yr)	53.5 (30–78, 14)
Sex (n, % female)	77, 80%
Education (n, % college education)	41, 43%
Income (n, % ≥ $40,000 year)	56, 59%
Race (n, % Caucasian)	91, 95%
MS Type (n, % RRMS)	79, 82%
Time since diagnosis (yr)	9 (1–43, 12)

Note. Values for age and disease duration are median (range, IQR). All other values are N, percentage. MS = multiple sclerosis. RRMS = relapsing-remitting multiple sclerosis. PDDS = Patient Determined Disease Steps scale; EDSS = Expanded Disability Status Score.

### Descriptive statistics for EDSS, PDDS, ambulatory, functional, and cognitive outcomes

The descriptive characteristics of the variables are provided in Table 2. The median PDDS and EDSS scores were 3.0 and 4.5, respectively: the scores are indicative of moderate disability with onset of gait impairment. The mean scores from the other measures are consistent with previous research involving persons with MS who have moderate disability [20-27].

**Table 2:** EDSS, PDDS, ambulatory, functional and cognitive characteristics of the 96 patients with multiple sclerosis

**Variable**	**Median (range, IQR)**
EDSS	4.5 (2-6.5, 3.0)
PDDS	3.0 (0-6, 3.0)
T25FW (s)	6.0 (3.1-24.5, 3.6)
TUG (s)	8.0 (3.3-33.5, 5.3)
6 MW (m)	424.6 (59.6-773.6, 189.3)
MSWS-12	49.0 (0-93.8, 44.8)
LL-FDI (BLEF)	20.0 (8-25, 8.0)
LL-FDI (ALEF)	11.5 (5-25, 8.8)
LL-FDI (UEF)	20.0 (8-25, 7.0)
Accelerometry (steps/day)	3630 (419-13136, 3635)
PASAT	43.0 (0.0-60.0, 19.8)
SDMT	44.5 (15.0-79.0, 13.0)

Note. IQR = Inter Quartile Range , 6 MW – Six-minute Walk, T25FW – Timed-25 Foot Walk, TUG – Timed Up and Go, MSWS-12 – Multiple Sclerosis Walking Scale, LLFDI – Late-Life Function and Disability Instrument, UEF – Upper Extremity Function, BLEF – Basic Lower Extremity Function, ALEF – Advanced Lower Extremity Function, SDMT – Symbol Digit Modalities Test, and PASAT – Paced Auditory Serial Addition Test.

### Criterion validity: correlation between PDDS and EDSS

**Figure 1:**
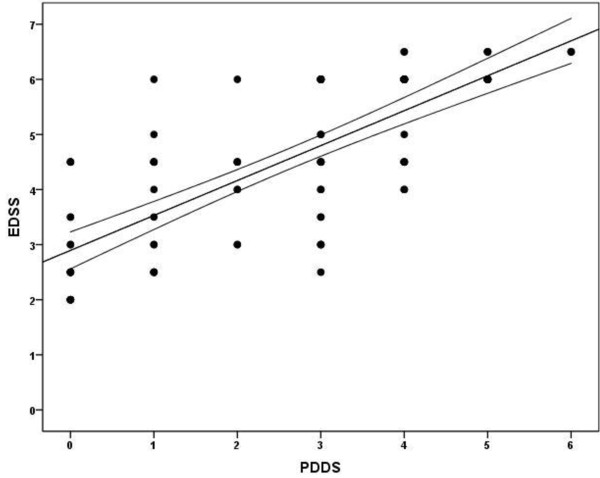
Scatterplot along with line of best fit and 95% confidence intervals of Expand Disability Status Scale (EDSS) and Patient Determined Disease Steps (PDDS) scores in 96 patients with multiple sclerosis.

Overall, there was a strong correlation between EDSS and PDDS scores (*ρ* = .783, 95% CI = .691-.850, *p* = .0001) and the scatterplot of scores is provided in Figure 1. We further regressed EDSS scores on PDDS scores (*F* (1,95) = 139.32, *p* < .001, *R*^2^ = .60), resulting in the regression equation, EDSS score = 2.9 + .63 (PDDS score). This indicates a strong linear association between scores, but that scores between scales are not isomorphic, for example, as a PDDS score of 0 corresponds with an EDSS score of 2.9. The correlation between EDSS and PDDS scores remained significant and strong within subsamples of mild (EDSS <4.5, n = 37) and moderate-to-severe (EDSS ≥4.5, n = 59) disability (mild: *ρ* = .641, 95% CI = .505-.746; moderate-to-severe: *ρ* = .688, 95% CI = .566-.781).

### Construct Validity: correlations between PDDS and EDSS with FS scores

The correlation coefficients and 95% confidence intervals between PDDS and EDSS with FS scores from the EDSS are provided in Table 3. Both PDDS and EDSS scores were strongly correlated with Pyramidal and Cerebellar FS scores, and moderately correlated with Sensory and Bowel/Bladder FS scores. The correlations were generally small between EDSS and PDDS with Visual, Mental, and Brainstem FS scores. Importantly, there were no significant differences in the correlations between the EDSS and PDDS scores with FS scores, based on the *p*-value from Fisher’s *z*-test.

**Table 3:** Correlations among EDSS and PDDS with functional system scores from the EDSS in 96 patients with multiple sclerosis

**Functional system**	**EDSS**	**EDSS 95% CI**	**PDDS**	**PDDS 95% CI**	**P-value**
Visual	0.369**	(0.182) - (0.530)	0.253*	(0.055) – (0.432)	.38
Brainstem	0.157	(-0.045) - (0.347)	0.184	(-0.017)- (0.371)	.85
Pyramidal	0.647**	(0.513) - (0.750)	0.578**	(0.427) – (0.698)	.45
Cerebellar	0.528**	(0.366) - (0.659)	0.501**	(0.334) – (0.637)	.80
Sensory	0.398**	(0.215) - (0.554)	0.392**	(0.208) – (0.549)	.96
Bladder/Bowel	0.317**	(0.124) - (0.487)	0.377**	(0.191) – (0.537)	.64
Mental	0.255*	(0.057) - (0.433)	0.169	(-0.033) – (0.357)	.54

Note. EDSS = Expanded Disability Status Scale, PDDS = Patient Determined Disease Steps scale, P-value for the statistical significance of the difference between correlation coefficients for EDSS and PDDS with FS scores, * = p < .05, ** = p < .01, CI = Confidence Interval.

### Construct validity: correlations between PDDS and EDSS with demographic, ambulatory, functional, and cognitive outcomes

The correlation coefficients for PDDS and EDSS with clinical, demographic, ambulatory, function, and cognitive variables are provided in Table 4. Both EDSS and PDDS scores were strongly correlated with ambulatory assessments (i.e., 6 MW, T25FW, TUG, Steps/day, & MSWS-12) and assessments of basic and advanced lower extremity functional (i.e., LL-FDI; BLEF & ALEF subscales). Both the EDSS and the PDDS correlated moderately with age, upper extremity function, and cognitive performance on SDMT. EDSS and PDDS scores generally had small correlations with MS disease duration, education, income, and cognitive performance on PASAT. Importantly, there were no significant differences in the correlations between the EDSS and PDDS with clinical, demographic, ambulatory, functional, and cognitive outcomes, based on the *p*-value from Fisher’s *z*-test.

**Table 4:** Correlations among scores from the EDSS and PDDS with demographic, ambulatory, functional and cognitive outcomes in 96 patients with multiple sclerosis

**Variable category**	**Measure**	**EDSS**	**EDSS 95% CI**	**PDDS**	**PDDS 95% CI**	**P-value**
Clinical/Demographic	Disease duration	0.199*	(-0.002) - (0.384)	0.202*	(0.002) - (0.387)	.98
	Age	0.446**	(0.270) - (0.593)	0.280**	(0.084) - (0.455)	.19
	Education	-0.352**	(-0.516) - (-0.163)	-0.274**	(-0.450) - (-0.078)	.56
	Income	-0.267**	(-0.444) - (-0.070)	-0.301**	(-0.473) - (-0.107)	.80
Ambulation	6 MW	-0.805**	(-0.866) - (-0.721)	-0.704**	(-0.793) - (-0.586)	.11
	T25FW	0.727**	(0.616) - (0.809)	0.627**	(0.488) - (0.735)	.20
	TUG	0.781**	(0.688) - (0.849)	0.717**	(0.603) - (0.802)	.32
	Accelerometry	-0.717**	(-0.802) - (-0.603)	-0.740**	(-0.819) - (-0.633)	.74
	MSWS-12	0.729**	(0.619) - (0.811)	0.801**	(0.715) - (0.863)	.23
Function	LL-FDI, BLEF	-0.715**	(-0.801) - (-0.601)	-0.748**	(-0.825)- (-0.644)	.63
	LL-FDI, ALEF	-0.727**	(-0.809) - (-0.616)	-0.739**	(-0.818) - (-0.632)	.86
	LL-FDI, UEF	-0.392**	(-0.549) - (-0.208)	-0.445**	(-0.593) -(-0.268)	.66
Cognition	SDMT	-0.404**	(-0.559) - (-0.221)	-0.409**	(-0.563) - (0.227)	.97
	PASAT	-0.261*	(-0.439) - (-0.064)	-0.244*	(-0.424) - (-0.046)	.90

Note. EDSS = Expanded Disability Status Scale, PDDS = Patient Determined Disease Steps scale, P-value for the statistical significance of the difference between correlation coefficients, 6 MW – Six-minute walk, T25FW – Timed-25 Foot Walk, TUG – Timed Up and Go, MSWS-12 – Multiple Sclerosis Walking Scale, LL-FDI – Late-Life Function and Disability Instrument, BLEF – Basic Lower Extremity Function, ALEF – Advanced Lower Extremity Function, UEF – Upper Extremity Function, SDMT – Symbol Digit Modalities Test, and PASAT – Paced Auditory Serial Addition Test, * = p < .05, ** = p < .01.

## Discussion

The present study examined the association between PDDS and EDSS scores and the associations between PDDS and EDSS scores with FS scores, ambulatory outcomes, cognitive processing speed, and clinical and demographic factors in 96 persons with MS. Overall, the PDDS had a strong, albeit not perfect, correlation with the EDSS, supporting criterion aspects of validity. The pattern and magnitude of correlations with FS scores, ambulatory outcomes, cognitive processing speed, and clinical and demographic variables further did not differ between the PDDS and EDSS, and supported construct aspects of validity. The magnitude and pattern of correlations between PDDS and EDSS scores was consistent between persons with mild and moderate-to-severe disability. Such results provide evidence for the validity of PDDS scores as a PRO of disability in persons with MS. The findings and limitations of this study do not suggest that the PDDS should replace the EDSS in clinical research, but rather that researchers and clinicians might consider the PDDS as an alternative assessment of disability, particularly when the EDSS is impractical (e.g., non-face-to-face research, lack of a clinician or other trained personnel available for administration), too costly, or inconvenient (e.g., time constraints of data collection, community-based research). Such recommendations and results are consistent with the original intention of the PDDS serving as a surrogate measure for the EDSS in clinical research involving MS.

This study examined the pattern of associations between FS scores with both the EDSS and PDDS in a cross-sectional analysis. Such an analysis is important for understanding the main components of neurological functioning that correlate with PDDS scores, and if the pattern is consistent between the PDDS and EDSS. The EDSS and PDDS had strong correlations with Pyramidal and Cerebellar FS scores, and moderate correlations with Sensory and Bowel/Bladder FS scores. The correlations were generally small between EDSS and PDDS with Visual, Mental, and Brainstem FS scores. Importantly, there were no differences in the magnitude of correlations between the EDSS and PDDS scores with FS scores. This pattern of correlations is generally consistent with previous research examining the associations between FS and EDSS scores in persons with MS [32,33] and suggests that motor involvement is a primary contributor to PDDS and EDSS scores. This supports the convergent aspects of construct validity for the PDDS.

The present study further examined the association between PDDS scores and measures of ambulation as well as demographic/clinical variables, cognition and functional limitations. This was warranted as the PDDS was developed based on the DS and this latter measure was primarily designed based on ambulation and motor functioning being the main determinants of disability in MS [3,4]. To that end, analyses indicated that the PDDS was most strongly correlated with ambulatory assessments (i.e., 6 MW, T25FW, TUG, Steps/day, & MSWS-12) and assessments of basic and advanced lower extremity functional limitations (i.e., LL-FDI scores). Other researchers too have reported associations between PDDS scores and MSWS-12 scores (*ρ = .847)*, 6 MW performance (*ρ = -.427)*, and free-living accelerometry (*ρ = .519)* in persons with MS [18]. Collectively, such results along with the pattern of associations with FS scores from the EDSS imply good convergent aspects of construct validity for the PDDS.

By comparison, the PDDS correlated moderately with age, upper extremity functional limitations on the LL-FDI, and performance on the SDMT, and weakly with MS disease duration, education, income, and performance on the PASAT. Importantly, the EDSS and PDDS correlated similarly with each of those outcomes. Such evidence alongside the weaker correlations between PDDS and EDSS with Visual, Mental, and Brainstem FS scores supports the divergent aspects of construct validity for the PDDS in persons with MS. Of further interest herein is the moderate relationship between cognitive processing speed (SDMT) and the EDSS and PDDS. Future studies might consider examining if impairment of cognitive processing speed influences the validity of PDDS and EDSS scores.

The primary benefit of the evidence provided in the current study is the provision of information on the PDDS as a valid PRO of disability in persons with MS. This is essential for building a stronger body of evidence regarding the actual validity of PDDS scores and clarifying misreporting of validity evidence in the literature. Indeed, we identified a correlation of .783 (95% CI = .691-.850) between PDDS and EDSS scores in the present study, and this is substantially and significantly less than the values of .93 and .958 often cited in the literature for the validity of PDDS scale; we believe, as noted in the introduction, that these large values may be unsubstantiated or reflect the association between EDSS and DS. Our correlation is stronger than the value of .64 reported in a validation study of the Performance Scales, another self-report of disability in MS [7]. Importantly, participants in the earlier study [7] were less disabled (median EDSS = 3.5) compared with the current sample (median EDSS = 4.5) and this may have accounted for the difference in correlations between studies. Indeed, analyses within disability subgroups in the present study support this as a likely explanation (i.e., the correlations were weaker, albeit still significant and strong, in the subgroups who would have a truncated range of scores). Overall, this study provides the first comprehensive assessment of the validity of PDDS scores as a PRO of disability in persons with MS.

There are multiple limitations of the present study. The first limitation is that we only provide validity evidence from a cross-sectional analysis, rather than data on the correspondence between changes in EDSS and PDDS over time. It will be important to perform such a longitudinal study, and doing so will allow for determination of the test-retest reliability of the PDDS over time. Although persons with a wide range of disability were included in this study, the results are limited in that our sample did not cover the full disability range present in persons with MS; there were no persons in the present analysis with an EDSS score of less than 2. We further did not include the DS in this study for an overall comparison of associations among EDSS, DS, and PDDS scores together and with other outcomes. This would have provided more definitive evidence on the source of misreporting regarding the validity of PDDS scores. This should be done as part of a longitudinal study. Lastly, the sample consisted mostly of Caucasian women with RRMS and a short disease-duration, and our results might not be generalizable broadly amongst those with MS. We only examined the validity of an English, print version of the PDDS, and future research should validate the PDDS in different languages and cultures as well as using electronic media (e.g. applications on cellular phones, the Internet, or tablets in the office or at home).

## Conclusions

Overall, this study provides novel and comprehensive information supporting the validity of the PDDS as a PRO of disability in persons with MS. The PDDS is an alternative to other, more complex, self-reported versions of the EDSS that have correlated well with the classical EDSS [34], but that still require significant amounts of time and have items that can be confusing for participants. Researchers could adopt the PDDS in clinical research and practice involving persons with MS alongside the EDSS or when the EDSS is impractical, too costly, or inconvenient. The PDDS scale is simple, economical, and efficient compared with the EDSS and DS and offers a potentially useful PRO of disability for clinical research and practice in MS.

## Competing interests

This project was funded by an investigator initiated grant from Biogen Idec. YCL was the recipient of a Du Pré Award from the Multiple Sclerosis International Federation.

## Authors’ contributions

RWM and DC acquired funding. RWM and BMS initiated the overall study and oversaw all data collection alongside JHP. YCL and RWM undertookstatistical analysis. YCL wrote the draft manuscript, RWM, JHP, BMS and DC revised the draft manuscript. All authors gave approval of the final versions.

## Pre-publication history

The pre-publication history for this paper can be accessed here:

http://www.biomedcentral.com/1471-2377/13/37/prepub
